# Supplementary material for: Ginkgolic acid attenuates echinococcus granulosus infection-induced hepatic fibrosis by inhibiting Smad4 SUMOylation
Source: PLoS Negl Trop Dis. 2026 Jan 13;20(1):e0013497. doi: 10.1371/journal.pntd.0013497 (PMC12818747; doi:10.1371/journal.pntd.0013497)
Supplement: S1 Table — (DOCX) [file pntd.0013497.s003.docx]

**S1 Table. Antibodies information used in immunohistochemicaly analysis**

| Antibodies | Company | Code | Dilution |
| --- | --- | --- | --- |
| Ubc9 | Abways | CY5571 | IHC (1∶800) |
| SUMO1 | Abcam | Ab32058 | IHC (1∶250) |
| SENP1 | Abways | CY1287 | IHC (1∶600) |
| α-SMA | Boster | BM0002 | IHC (1∶800) |
| COL1A1 | Cell Singaling Technology | #72026 | IHC (1∶800) |
