# Supplementary material for: Ginkgolic acid attenuates echinococcus granulosus infection-induced hepatic fibrosis by inhibiting Smad4 SUMOylation
Source: PLoS Negl Trop Dis. 2026 Jan 13;20(1):e0013497. doi: 10.1371/journal.pntd.0013497 (PMC12818747; doi:10.1371/journal.pntd.0013497)
Supplement: S2 Table — (DOCX) [file pntd.0013497.s004.docx]

**S2 Table. Antibodies information used in tissue immunofluorescence analysis**

| Antibodies | Company | Code | Dilution |
| --- | --- | --- | --- |
| SUMO1 | Abcam | Ab32058 | IF-P (1∶250) |
| F4/80 | Abcam | Ab300421 | IF-P (1∶5000) |
| iF488-Tyramide | servicebio | G1231-25UL | IF -P (1∶500) |
| iF555-Tyramide | servicebio | G1233-25UL | IF-P (1∶500) |
