# Supplementary material for: Ginkgolic acid attenuates echinococcus granulosus infection-induced hepatic fibrosis by inhibiting Smad4 SUMOylation
Source: PLoS Negl Trop Dis. 2026 Jan 13;20(1):e0013497. doi: 10.1371/journal.pntd.0013497 (PMC12818747; doi:10.1371/journal.pntd.0013497)
Supplement: S3 Table — (DOCX) [file pntd.0013497.s005.docx]

**S3 Table. Primer sequences for qRT-PCR**

| Symbol | Forward | Reverse |
| --- | --- | --- |
| SENP1 | GTTCCGGTTCGGACTTTGTA | GTGGCAGGAGGTGGGTTTT |
| β-actin | AACCGCGAGAAGATGACCCAG | GGATAGCACAGCCTGGATAGCAA |
