# Supplementary material for: Ginkgolic acid attenuates echinococcus granulosus infection-induced hepatic fibrosis by inhibiting Smad4 SUMOylation
Source: PLoS Negl Trop Dis. 2026 Jan 13;20(1):e0013497. doi: 10.1371/journal.pntd.0013497 (PMC12818747; doi:10.1371/journal.pntd.0013497)
Supplement: S4 Table — (DOCX) [file pntd.0013497.s006.docx]

**S4 Table. Antibodies information used in Western blot analysis.**

| Antibodies | Company | Code | Dilution |
| --- | --- | --- | --- |
| Ubc9 | Cell Singaling Technology | #4786 | WB (1∶1000) |
| SUMO1 | Abcam | Ab32058 | WB (1∶1000) |
| SENP1 | Cell Singaling Technology | #11929 | WB (1∶1000) |
| α-SMA | Cell Singaling Technology | #48938 | WB (1∶1000) |
| COL1A1 | Cell Singaling Technology | #72026 | WB (1∶1000) |
| Smad4 | Proteintech | 10231-1-AP | WB (1∶1000) |
| CD206 | Cell Singaling Technology | #24595 | WB (1∶1000) |
| CD86 | Cell Singaling Technology | #91882 | WB (1∶1000) |
| PCNA | Boster | BM0104 | WB (1∶1000) |
| β-actin | Zhongshan Bridge | TA-09 | WB (1∶5000) |
| GAPDH | Zhongshan Bridge | TA-08 | WB (1∶2000) |
| Histone H3 | Boster | A12477-2 | WB (1∶1000) |
| HRP* Goat Anti Rabbit IgG | Zhongshan Bridge | ZB-2301 | WB (1∶10000) |
| HRP* Goat Anti Mouse IgG | Zhongshan Bridge | ZB-2305 | WB (1∶10000) |
