# Supplementary material for: Ginkgolic acid attenuates echinococcus granulosus infection-induced hepatic fibrosis by inhibiting Smad4 SUMOylation
Source: PLoS Negl Trop Dis. 2026 Jan 13;20(1):e0013497. doi: 10.1371/journal.pntd.0013497 (PMC12818747; doi:10.1371/journal.pntd.0013497)
Supplement: S5 Table — (DOCX) [file pntd.0013497.s007.docx]

**S5 Table. Antibodies information used in cellular immunofluorescence assay**

| Antibodies | Company | Code | Dilution |
| --- | --- | --- | --- |
| SUMO1 | Abcam | Ab32058 | IF (1∶2000) |
| Smad4 | Proteintech | 10231-1-AP | IF (1∶2000) |
| iF488-Tyramide | servicebio | G1231-25UL | IF (1∶500) |
| iF555-Tyramide | servicebio | G1233-25UL | IF (1∶500) |
