# Supplementary material for: Ginkgolic acid attenuates echinococcus granulosus infection-induced hepatic fibrosis by inhibiting Smad4 SUMOylation
Source: PLoS Negl Trop Dis. 2026 Jan 13;20(1):e0013497. doi: 10.1371/journal.pntd.0013497 (PMC12818747; doi:10.1371/journal.pntd.0013497)
Supplement: Attachment — Submitted filename: Response to Reviewers.docx [file pntd.0013497.s009.docx]

Robert Adamu SHEY, Ph.D.
Guest Editor
PLOS Neglected Tropical Diseases

December, 4, 2025

Dear Robert Adamu SHEY, Ph.D.,

I would like to submit a revised manuscript for publication in PLOS Neglected Tropical Diseases, titled “Ginkgolic acid attenuates *echinococcus granulosus*  infection-induced hepatic fibrosis by inhibiting Smad4 SUMOylation” (Manuscript No. PNTD-D-25-01450 )

Thank you very much for your letter, questions and the reviewers’ comments which we found very helpful in improving our manuscript. We have revised the paper according to the comments in a point-by-point format as shown below.

The revised sections and sentences are blue colored especially in parts of introduction, methods, results and discussion.

In addition, all authors declare no conflicts of interest to report.

We hope the revised manuscript is satisfactory and suitable for publication in your journal. Please let us know if further revisions are needed.

Sincerely yours,

Xueling Chen, Ph.D.

Shihezi University School of Medicine

North 4th Road

Shihezi, Xinjiang China 832003

Phone :+86-138-9950-4062

E-mail: [chenxueling@shzu.edu.cn](mailto:chenxueling@shzu.edu.cn)

**Reviewer #1:**

This manuscript investigates the therapeutic potential of GA in alleviating liver fibrosis induced by Echinococcus granulosus infection, focusing on its inhibition of Smad4 SUMOylation and modulation of macrophage–hepatic stellate cell crosstalk. The study is well-structured, combining clinical samples, animal models, and in vitro experiments to comprehensively explore the molecular mechanism.

While the findings are promising and suggest GA as a potential anti-fibrotic agent, the manuscript requires revisions to enhance clarity, statistical rigor, and methodological detail.

**Response:** Thank you for your careful review of this manuscript. We have made the following modifications one by one to the article according to your suggestions and requirements.

For the Introduction Section

While the introduction touches upon multiple concepts—CE, GA, SUMOylation, macrophage polarization, and TGF-β/Smad signaling—the connections between these ideas are not seamlessly established. Although the research gap is mentioned, it should be more explicitly stated what specific unanswered question the study aims to address.

**Response:** Thank you for your valuable suggestions regarding the introduction section, which is crucial for enhancing the logic and clarity of our manuscript. We have adjusted and optimized the previously scattered concepts, starting from the clinical pathological issue (CE hepatic fibrosis), moving to the core cellular events (HSC activation and M2 polarization of macrophages), then to the molecular mechanism (SUMOylation‑mediated regulation of Smad4 function), and finally introducing the potential therapeutic exploration with GA. Furthermore, we have proposed a scientific hypothesis at the end, introduced the research content, and clarified the significance of the study.

For the Methods Section
The authors please provide missing critical details regarding experimental procedures, statistical reporting, and cell culture conditions.

**Response:** We are grateful to the reviewers for their detailed comments on the methodology section. We have made the following additions and improvements to the description of the experimental methods based on your suggestions:

1. We have supplemented the extraction steps and activity identification methods for the hydatid cyst fluid and protoscoleces, as well as the transfection process of SENP1 siRNA, and the experimental system and primer sequences for qRT-PCR.
2. We have separated the previously combined descriptions of "histopathology, immunohistochemistry and immunofluorescence staining" into independent experiments of "Histopathological and immunohistochemical Staining", "Tissue Immunofluorescence Assay" and "Cellular Immunofluorescence Assay", and have supplemented the key experimental parameters for each, including antibody sources and image acquisition.
3. We have reorganized the conditioned medium co-culture experiment of macrophages and HSCs, clearly explaining the experimental groups, treatment conditions, time points and detection indicators.
4. Supplemented the Western blot methodology with information on key antibodies and the grayscale quantification method used for analysis.
5. Supplemented the description of the sample size (n value) of the animal experiments, the number of independent repetitions of the cell experiments, as well as the specific statistical test methods and data presentation forms used in all relevant figure legends.

For the Results Section
The language often implies a direct and proven causal relationship where the data primarily show correlation. The narrative flow between some paragraphs is abrupt.
the Results section must be substantially revised to include essential statistical details, employ more precise and cautious language regarding mechanistic claims, and improve overall clarity and language quality.
**Response:** Thank you for your profound and detailed review comments on the results section. In response to your suggestions, we have made the following revisions to the results section:

1. Optimized language expression: We systematically corrected the wording in the results section, replacing the previous expressions that might imply direct causal relationships (such as "resulted in" or "therefore proves") with more precise and cautious descriptive language (such as "related to..." or "accompanied by... changes"), to accurately reflect the correlations presented by the data.
2. Strengthened logical connection: We optimized the beginning and ending of each result subsection, as well as the transitional sentences between paragraphs. This made the various results form a coherent and progressive scientific narrative.
3. In the figure legends of the relevant results, we supplemented statistical information, including the specific sample size (n value), precise P-values or significance level annotations (*p < 0.05, **p < 0.01, etc.), and unified and optimized the presentation form of the data (such as mean ± standard error).

For the Discussion Sections
While a limitations paragraph is included, it remains superficial. The authors should delve deeper: Pharmacokinetics/Pharmacodynamics；Specificity of GA；In vivo validation.

**Response:**Thank you for your profound and constructive comments on the discussion section. Based on the suggestions you provided (pharmacokinetics/pharmacodynamics, specificity of GA, in vivo validation), we have optimized the "Limitations" section as follows:

1. Optimization for pharmacokinetics/pharmacodynamics (PK/PD): "in terms of pharmacokinetics, the *in vivo* course of GA in infection models has not been defined. Although the existing dosing regimens refer to previous studies and simulate the clinical treatment window, their local distribution, metabolism and effective duration of action still need to be systematically evaluated, which is crucial to optimize the dosing strategy and promote the clinical translation." We added "quantitative assessment of which key parameters and their significance."
2. Deepening of GA specificity: After modification, we first indicated the complexity of GA as a multi-target natural product, and then pointed out that although the SENP1 knockdown experiment suggested its involvement, it still cannot completely rule out the possibility that GA indirectly affects SUMOylation through other parallel pathways (such as direct anti-inflammatory). We further proposed that a SUMOylation site mutant Smad4 cell model could be used to establish its specificity.
3. Deepening of in vivo validation of in vitro mechanisms: After modification, we first explained that animal experiments at the tissue level (such as reversing SUMOylation protein expression, reducing SUMO1⁺ macrophage infiltration) provided indirect support for the in vitro mechanisms, but currently there is a lack of evidence for directly verifying the key steps of the "GA-SENP1-Smad4 SUMOylation" regulatory axis in vivo, on specific cell types (such as macrophages). We proposed that future studies could be conducted through knockout of mice or spatial multi-omics for more in-depth analysis.

Best regards

Xueling Chen

**Reviewer #2:** In the animal experiment, the purpose of the "GA safety group" is unclear; it is recommended to clarify its role as a drug toxicity control group.Additionally, detailed justification for initiating GA administration at the 8th week is lacking.
The mechanism by which GA inhibits Smad4 SUMOylation in Figure 4 lacks direct evidence (e.g., SENP1 overexpression/knockdown experiments).
Control experiments demonstrating GA's specificity in inhibiting SUMOylation are absent (e.g., comparison with other SUMO inhibitors).

**Response:**Thank you very much for your specific and constructive comments on this manuscript. Each of your suggestions pointed out key areas in our study that required strengthening and refinement, which is of great value for enhancing the scientific rigor and logical integrity of the paper. Following your guidance, we have made the following revisions and improvements in the corresponding sections of the manuscript:

1. **Description of the purpose of the "GA safety group"**

In Result 2, we stated that the setup of the "GA safety group" (i.e., uninfected mice that only received GA treatment) was intended as a drug toxicity control group to assess the potential impact of GA on normal organs. The relevant description has been revised as: "Moreover, histologic examination of major organs from the GA-only control group showed no significant differences from the normal control group (S2 Fig), indicating no observable organ toxicity under the experimental conditions." This clarifies that the role of this group is to eliminate the interference of the drug's own toxicity on the interpretation of therapeutic efficacy.

**2. Explanation of the reasons for granting GA starting from the 8th week**

This protocol is based on the previous study of our team (Wang E, Liao Z, Wang L, Liao Y, Xu X, Liu P, et al. A combination of pirfenidone and TGF-β inhibition mitigates cystic echinococcosis-associated hepatic injury. Parasitology. 2021;148(7):767-778. https://doi.org/10.1017/S0031182021000287 PMID: 33583470), that is, in a mouse model with 3,000 PSCs injected under the liver capsule, the initial formation of cysts accompanied by early fibrosis could be observed at the 8th week after infection. The intervention initiated at this point aims to simulate the early clinical treatment window, thereby making the research more relevant for translational reference.

**3. Supplemental direct evidence for GA‑mediated inhibition of Smad4 SUMOylation in Fig. 4**

In Result 4, we have supplemented the data with SENP1 knockdown experiments. The added text states: “To clarify the role of SENP1 in this process, we knocked down SENP1 expression in THP-1 cells. The results showed that SENP1 knockdown attenuated the EgCF-induced Smad4 SUMOylation, while the ability of GA to reverse this effect was significantly enhanced, indicating that the upregulation of SENP1 is a critical mechanism by which GA inhibits Smad4 SUMOylation (Fig 4h). ” This experiment strengthens the causal relationship within the “GA → SENP1 → Smad4 SUMOylation” regulatory axis.

**4. Future research directions on the specificity of GA's action**

In the "Limitations" section of the discussion part, we pointed out that this study lacks the specific validation of GA's inhibition of SUMOylation. We have listed this as a current deficiency in the research and regard it as an important direction for future research, in order to further clarify the target and mechanism specificity of GA's action.

Best regards

Xueling Chen
